# Development of a Risk Model to Identify and Prevent Factors Influencing Erectile Dysfunction After Robotic Radical Prostatectomy

**DOI:** 10.3390/jcm14144903

**Published:** 2025-07-10

**Authors:** Hakan Karaca, Resul Sobay, Metin Mod, Ahmet Tahra, Hasan Samet Güngör, Abdurrahman İnkaya, Eyüp Veli Küçük

**Affiliations:** 1 Department of Urology, Ardahan State Hospital, 75000 Ardahan, Turkey; 2 Department of Urology, Health Science University Umraniye Training and Research Hospital, 34764 Istanbul, Turkey; drresulsobay@gmail.com (R.S.); ahmettahra@gmail.com (A.T.); drsametgngr@gmail.com (H.S.G.); ainkaya@hotmail.com (A.İ.); eyupveli@hotmail.com (E.V.K.); 3 Department of Urology, Basaksehir Cam Sakura City Hospital, 34480 Istanbul, Turkey; metinmod41@gmail.com

**Keywords:** erectile dysfunction, prediction model, prostate cancer, robotic radical prostatectomy

## Abstract

**Background/Objectives:** Prostate cancer ranks as the second-most prevalent cancer globally, and is the fifth-ranking cause of cancer-related mortality. Radical prostatectomy presents a significant risk of postoperative sequelae, including erectile dysfunction. Postoperative erectile dysfunction adversely affects the patient’s quality of life and can severely impact total treatment satisfaction. Nomograms have demonstrated efficacy in forecasting diverse outcomes in urology. We sought to create a nomogram to facilitate a more precise, evidence-based, and individualized prediction of erectile function outcomes following radical prostatectomy. Between January 2018 and January 2022, one hundred and eleven prostate cancer patients had robot-assisted radical prostatectomy, excluding those who had undergone prior transurethral prostatectomy, radiotherapy, or hormone therapy. Demographics, medical records, preoperative and postoperative erectile function statuses, and IIEF scores (≥17 indicating retained erections, <17 indicating full erectile dysfunction) were evaluated. **Outcomes**: Patients’ ages ranged from 45 to 76 years, with an average of 61.18 ± 6.72 years. Patients in the emergency department were considerably older (*p* = 0.004; *p* < 0.01) and exhibited elevated Charlson Comorbidity Indices (3.63 ± 0.85; *p* = 0.004; *p* < 0.01). Preoperative IIEF scores in ED patients were lower (14.29 ± 5.34), although obturator internus thickness (20.61 ± 2.91) and intraprostatic urethra length (36.48 ± 9.3) were considerably elevated. Altered surgical techniques were linked to maintained erections (*p* = 0.002; *p* < 0.01), but traditional approaches were connected with erectile dysfunction (*p* = 0.007; *p* < 0.01). Bilateral nerve-sparing procedures were more prevalent among patients preserving erectile function (*p* = 0.003; *p* < 0.01). **Conclusions**: The nomogram, which includes age, Charlson Comorbidity Index, preoperative IIEF, obturator internus thickness, intraprostatic urethra length, surgical technique, and degree of nerve preservation, provides clinicians with a pragmatic instrument for forecasting postoperative erectile dysfunction in prostate cancer patients.

## 1. Introduction

Prostate cancer ranks as the second-most prevalent cancer in men globally, with approximately 1.4 million diagnoses reported in 2020. It is the fifth-ranking cause of cancer-related mortality, contributing to an estimated 375,000 deaths [1]. Incidence and mortality rates exhibit considerable variation across different geographic locations. Identifying risk factors for prostate cancer development is essential for primary and secondary prevention [2].

Radical prostatectomy (RP) is a widely utilized therapeutic approach for managing prostate cancer. This surgical approach results in positive outcomes in terms of disease-free and overall survival rates; however, it is linked to several potential complications, particularly postoperative erectile dysfunction (ED) and urinary incontinence. The declining average age at diagnosis has rendered the preservation and recovery of postoperative erectile function a critical issue in urology [3,4]. A diverse array of ED rates subsequent to RP has been documented. Tal et al. conducted a meta-analysis on ED rates post-RP, revealing significant variability in outcomes, with incidence rates between 14% and 90%. This extensive variability restricts the applicability of these findings in informing clinical decision-making and delivering effective postoperative counseling for patients with prostate cancer [5]. Mulhall similarly reported significant variability in the incidence of erectile dysfunction following radical prostatectomy, with rates between 12% and 96%. Higher ED rates were observed in multi-center, multi-surgeon studies, compared to those conducted at single centers by individual surgeons, indicating the impact of surgical technique and institutional factors on postoperative outcomes [6]. Resnick et al. reported findings from the Prostate Cancer Outcomes Study (PCOS), a population-based cohort study that prospectively tracked men diagnosed with prostate cancer over a 15-year period starting in 1991 [7]. In a study involving 1655 participants evaluated at baseline and at 2, 5, and 15 years after receiving either RP or radiation therapy, individuals who underwent prostatectomy exhibited a significantly higher incidence of ED at both the 2-year and 5-year marks post-treatment. During the follow-up periods, 78% to 87% of men reported experiencing erections inadequate for sexual intercourse [7]. Johansson found that among 173 men, 146 (84%) experienced ED following RP [8].

ED is assessed using the International Index of Erectile Function (IIEF) questionnaire. Patients with an IIEF score of 17 or higher are classified as having preserved erectile function, while those with a score below 17 are categorized as having complete erectile dysfunction.

The nomogram has demonstrated efficacy in predicting diverse outcomes within the field of urology [9,10]. Predictive models have demonstrated superiority over clinician estimations. The complication profile associated with a specific treatment strategy can impact the patient’s decision-making process, and the realistic expectations that develop can influence the patient’s satisfaction with the chosen treatment [11,12]. In this study, we sought to create a nomogram to facilitate more precise, evidence-based, and individualized predictions of erectile function (EF) outcomes following RP.

## 2. Materials and Methods

### 2.1. Compliance with Ethical Standards

Ethical approval for the study was obtained from the Ethics Committee of Health Science University Umraniye Research and Training Hospital, with approval number 0.01/275.

### 2.2. Study Design

The study comprised 111 patients who received robot-assisted radical prostatectomy (RARP) for prostate cancer carried out by a single surgeon between January 2018 and January 2022. All patients had no previous history of transurethral prostatectomy, radiotherapy, or hormone therapy. The demographic features, medical records, and preoperative and postoperative erectile function statuses of the patients were analyzed.

Multiparametric MRI assessments were conducted by two experienced urologists and subsequently evaluated in conjunction with a radiologist possessing substantial clinical expertise in urology.

Preoperative age, Charlson Comorbidity Index, preoperative IIEF score, preoperative smoking history, preoperative PSA levels, Gleason grade group in biopsy, prostate volume, presence of median lobe, bladder neck invasion, interval between biopsy and surgery, surgical technique employed, duration of surgery, extent of nerve preservation, perioperative blood loss, presence of lymph node dissection, thickness of obturator internus, length of membranous urethra, angle with prostate axis, intraprostatic urethra length, urethral width, postoperative pathological stage, positivity of postoperative surgical margins, duration of postoperative catheterization, early and late use and duration of PDE5 inhibitors postoperatively, and early and late application and duration of intracavernous vasoactive agents postoperatively were all assessed. A nomogram was developed utilizing criteria recognized as major indicators of the risk for postoperative erectile dysfunction.

During the postoperative phase, all patients were instructed to engage in penile rehabilitation using either daily oral tadalafil 5 mg or tadalafil 20 mg administered three times weekly after catheter removal. At the 6-month follow-up, patients who could not attain erections received intracavernosal injection of vasoactive drugs. The IIEF score was utilized to evaluate erection status at the 6-month follow-up.

All procedures in the study were conducted transperitoneally with the Da Vinci Xi robotic system. The nerve-sparing procedure was implemented in accordance with the previously described ultrapreservation anterior-sparing method [13].

### 2.3. Statistical Analysis

Statistical Package for the Social Sciences (SPSS) version 27 was utilized for statistical analysis. Descriptive statistical techniques (mean, standard deviation, median, frequency, percentage, minimum, maximum) were employed in the analysis of the study data. The normality of distribution for quantitative data was assessed utilizing the Shapiro–Wilk test and graphical analyses. For continuous variables with normal distribution, Student’s *t*-test was employed for comparisons between two groups. For continuous variables lacking normal distribution, the Mann–Whitney U test was utilized. For categorical variables, Pearson’s chi-square test was used when expected cell frequencies were adequate (≥5), while Fisher’s exact test or Fisher–Freeman–Halton test were employed when expected cell frequencies were inadequate (<5). One-way analysis of variance and Bonferroni-adjusted pairwise comparisons were employed for comparisons among multiple groups of normally distributed quantitative variables, whilst Student’s *t*-test was utilized for comparisons between two groups. For quantitative variables lacking a normal distribution, Kruskal–Wallis and Dunn–Bonferroni tests were employed for comparisons across many groups, whilst the Mann–Whitney U test was utilized for comparisons between two groups. The Wilcoxon signed-ranks test was utilized for within-group comparisons of quantitative variables lacking a normal distribution. Logistic regression analysis and nomograms were employed in multivariate evaluations. Statistical significance was defined as *p* < 0.05.

## 3. Results

In the initial year of the trial, out of 111 participating patients, 73 exhibited complete ED, whilst 38 maintained EF. All demographic and preoperative variables potentially influencing postoperative EF were assessed, revealing associations with age, Charlson Comorbidity Index, and IIEF score (Table 1).

The multiparametric MRI data of the patients were evaluated, revealing a link that suggests obturator internus muscle thickness and intraprostatic urethral length influence post-prostatectomy erectile function (Table 2).

In patients with intact erectile function, the adoption rate of the modified surgical technique—ultrapreservation anterior-sparing RARP—was markedly elevated (*p* = 0.002; *p* < 0.01). Implementation of the bilateral nerve-sparing approach resulted in a statistically significant enhancement in this group. (*p* = 0.003; *p* < 0.01) (Table 3).

A logistic regression analysis was performed to ascertain the risk factors affecting erectile dysfunction and to create a nomogram.

The impacts of age, preoperative IIEF score, obturator internus thickness, intraprostatic urethral length, Charlson Comorbidity Index, nerve-sparing technique, and surgical technique on erectile dysfunction were evaluated using logistic regression analysis.

The model exhibited statistical significance.

(Chi-Square = 56.038; *p* = 0.001; *p* < 0.01), with an explanatory power (Nagelkerke R^2^) of 82%.
**Model 1**Surgical TechniqueModified (Ultrapreservation Anterior-Sparing)Nerve-Sparing TechniqueBilateral
**Score**Preoperative IIEF ScoreCut Off: <13.1ODDS: 3.3**4.79**Obturator Internus Muscle ThicknessCut Off: ≥20.2ODDS: 1.7**2.47**Charlson Comorbidity Index Cut Off: ≥4ODDS: 1**1.45**Intraprostatic Urethral LengthCut Off: ≥35.8ODDS: 0.75**1.09**AgeCut Off: ≥62ODDS: 0.14**0.20**

**Score****10**

**Figure 1:**
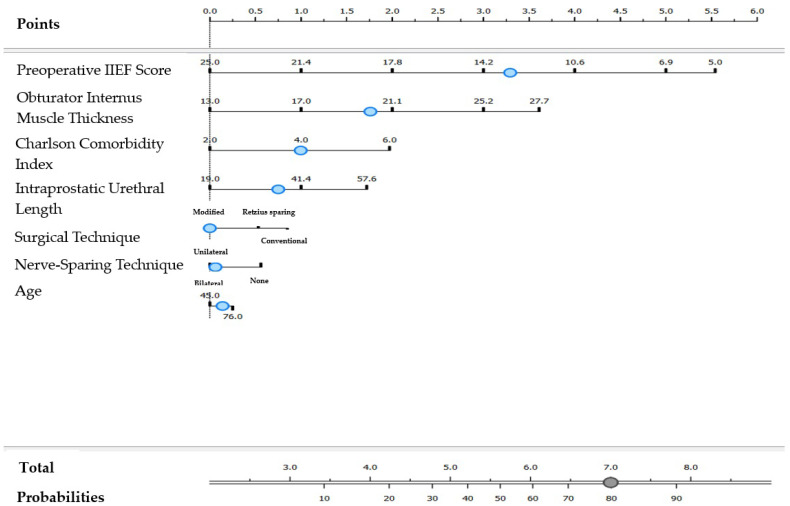
Nomogram model for complete erectile dysfunction in cases with modified surgical technique and nerve-sparing technique applied bilaterally. The blue dots represent the cut-off values displayed in the model. Complete erectile dysfunction = [Preoperative IIEF (<13.0)] × 4.79 + [Obturator Internus Thickness (≥20.2)] × 2.47 + [Charlson Comorbidity Index (≥4)] × 1.45 + [Intraprostatic Urethral Length (≥35.8)] × 1.09 + [Age (≥62)] × 0.20.

For patients undergoing a modified surgical technique combined with a bilateral nerve-sparing technique, the formula for predicting complete erectile dysfunction was as follows (Figure 1):
**Model 2**Surgical TechniqueModified

Nerve-Sparing TechniqueUnilateral
**Score**Preoperative IIEF ScoreCut Off: <13.1ODDS: 3.3**4.86**Obturator Internus Muscle ThicknessCut Off: ≥20.2ODDS: 1.7**2.43**Charlson Comorbidity Index Cut Off: ≥4ODDS: 1**1.43**Intraprostatic Urethral LengthCut Off: ≥35.8ODDS: 0.75**1.07**AgeCut Off: ≥62ODDS: 0.14**0.21**

**Score****10**

**Figure 2:**
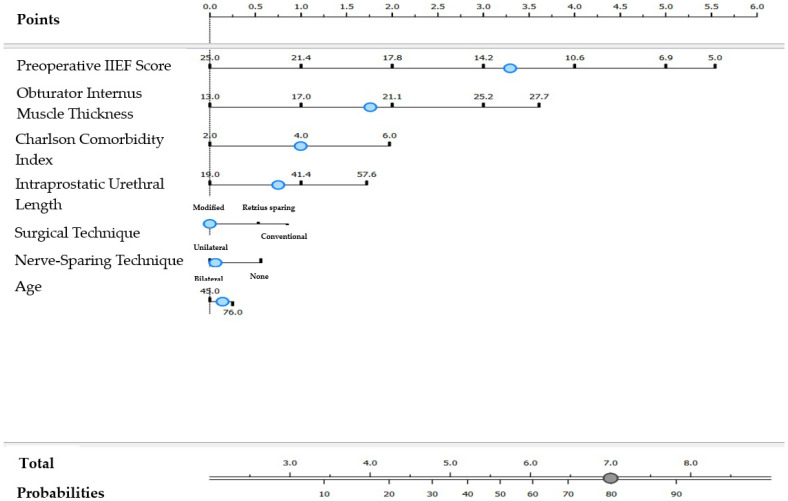
Nomogram model for complete erectile dysfunction in cases with modified surgical technique and nerve-sparing technique applied unilaterally. The blue dots represent the cut-off values displayed in the model.

For individuals undergoing a modified surgical technique with unilateral nerve-sparing approach, the nomogram for complete erectile dysfunction was as follows: [Preoperative IIEF (<12.9)] × 4.86 + [Obturator Internus Thickness (≥20.2 mm)] × 2.43 + [Charlson Comorbidity Index (≥4)] × 1.43 + [Intraprostatic Urethral Length (≥35.8 mm)] × 1.07 + [Age (≥62 years)] × 0.20 (Figure 2).
**Model 3**Surgical TechniqueConventional

Nerve-Sparing TechniqueBilateral
**Score**Preoperative IIEF ScoreCut Off: <15.9ODDS: 2.5**4.11**Obturator Internus Muscle ThicknessCut Off: ≥20.2ODDS: 1.76**2.79**Charlson Comorbidity Index Cut Off: ≥4ODDS: 1**1.64**Intraprostatic Urethral LengthCut Off: ≥35.8ODDS: 0.75**1.23**AgeCut Off: ≥62ODDS: 0.14**0.23**

**Score****10**

**Figure 3:**
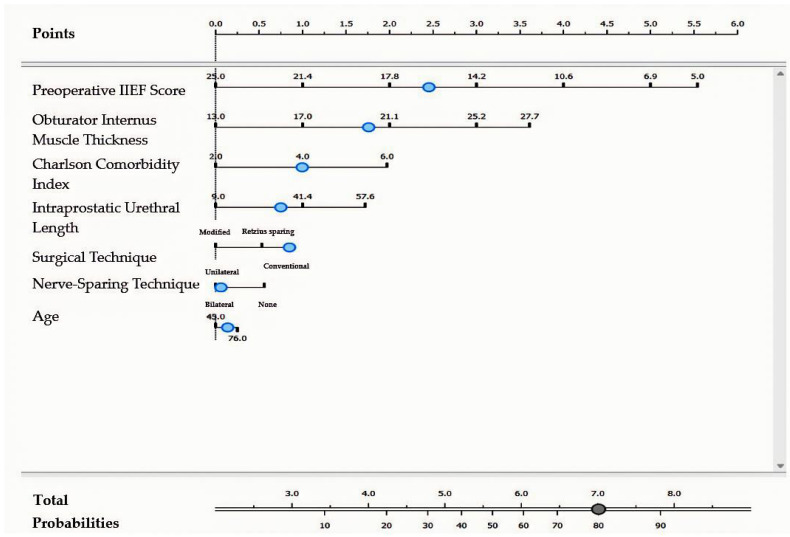
Nomogram model for complete erectile dysfunction in patients undergoing conventional surgical technique with bilateral nerve-sparing approach. The blue dots represent the cut-off values displayed in the model.

For patients who underwent a conventional surgical technique and bilateral nerve-sparing technique, the formula for complete erectile dysfunction was as follows: [Preoperative IIEF (<16.1)] × 4.11 + [Obturator Internus Thickness (≥20.2)] × 2.79 + [Charlson Index (≥4)] × 1.64 + [Intraprostatic Urethra Length (≥35.8)] × 1.23 + [Age (≥62)] × 0.23 (Figure 3).
**Model 4**


Surgical TechniqueConventional

Nerve-Sparing TechniqueUnilateral
**Score**Preoperative IIEF ScoreCut Off: <15.9ODDS: 2.5**4.11**Obturator Internus Muscle ThicknessCut Off: ≥20.2ODDS: 1.76**2.79**Charlson Comorbidity Index Cut Off: ≥4ODDS: 1**1.64**Intraprostatic Urethral LengthCut Off: ≥35.8ODDS: 0.75**1.23**AgeCut Off: ≥62ODDS: 0.14**0.23**

**Score****10**

**Figure 4:**
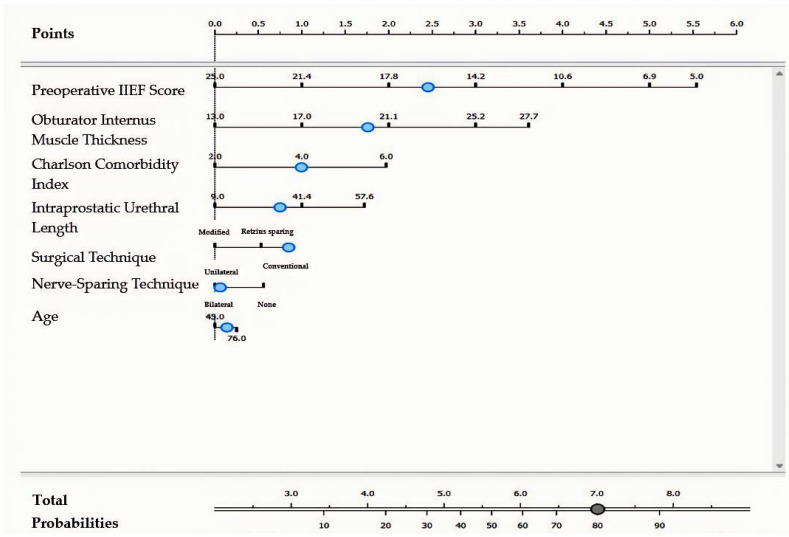
Nomogram model for complete erectile dysfunction in patients undergoing conventional surgical technique with unilateral nerve-sparing approach. The blue dots represent the cut-off values displayed in the model.

For patients who underwent a conventional surgical technique with unilateral nerve-sparing, complete erectile dysfunction was predicted by the following model: [Preoperative IIEF (<16.1)] × 4.11 + [Obturator Internus Thickness (≥20.2 mm)] × 2.79 + [Charlson Comorbidity Index (≥4)] × 1.64 + [Intraprostatic Urethral Length (≥35.8 mm)] × 1.23 + [Age (≥62 years)] × 0.23 (Figure 4).

## 4. Discussion

Prostate cancer ranks among the most prevalent cancers in the male population. The prevalence of screening methods has led to earlier-stage diagnoses of prostate cancer. The increasing incidence of prostate cancer in younger men has heightened concerns regarding post-cancer EF.

RP is the most commonly executed therapeutic approach for localized or locally progressed prostate carcinoma. RARP has emerged as a prevalent method in the surgical management of prostate cancer.

Due to the heterogeneity in EF outcomes documented in the literature, clinicians conducting preoperative consultations encounter a conundrum. The multitude of factors affecting post-prostatectomy EF, along with the intricate interrelations among these parameters, leads to variability in predicting erectile outcomes. Consequently, a nomogram that offers an evidence-based, individualized prediction would be exceedingly advantageous.

Brajtbord et al. documented erectile recovery following RP in two age categories: ≤60 years and >60 years. Older males exhibited an increased probability of clinically significant deterioration in EF [14]. Alemozaffar et al. reported in a study on a prediction model for postoperative ED that advancing age correlated with a diminished likelihood of maintaining EF, even after controlling for baseline performance [15]. In our study, consistent with prior research, we noted a rise in ED with increasing age. The results indicate that advancing age is a notable risk factor for ED.

Salter et al. demonstrated that a history of smoking may be linked to ED three months post-RP [16]. Our investigation, however, revealed no statistically significant difference in smoking rates among the cases based on IIEF scores. Variations in the timing of postoperative EF assessment, as well as in the duration and level of cigarette consumption, may account for discrepancies between studies. Given the rising popularity of electronic cigarette usage, future studies may investigate the effects of these products on erectile function post-RP.

The application of a modified surgical technique (ultrapreservation anterior-sparing RARP) was markedly more prevalent among patients who maintained EF (*p* = 0.002; *p* < 0.01), while the conventional technique was notably more common among individuals with complete ED (*p* = 0.007; *p* < 0.01).

Bhat et al. indicated that a greater extent of nerve sparing correlated with enhanced EF outcomes [17]. Zhao et al. indicated that postoperative potency levels, both early and late, were superior in patients who received intrafascial RP compared to those who received non-intrafascial RP [18]. In accordance with the existing literature, our investigation revealed that use of the bilateral nerve-sparing technique was much greater in instances of retained EF.

Our investigation revealed no statistically significant variations in Gleason grade groups based on IIEF levels. A study by Pikramenos et al. reported a potential correlation between high Gleason scores and preoperative IIEF levels; however, no statistically significant relationship with postoperative IIEF values was observed [19]. This result may be ascribed to the sample size. Research involving bigger cohorts may uncover statistically significant disparities.

The primary limitations of this study are its retrospective design and small sample size. Although RARP was performed by a single highly experienced surgeon (with over 1000 RARPs) in our study, we believe that implementation of RARP by various surgeons is warranted. Additionally, the single-center nature of our study may be regarded as a limitation.

A principal strength of our study is its comprehensive evaluation of all patient-related factors, surgical procedures, and parameters that may affect EF post-RP, with patients being meticulously tracked during the postoperative period. Before now, no studies in the literature have investigated the correlation between post-prostatectomy ED and factors such as membranous urethral length, the angle between the membranous urethra and the prostate axis, obturator internus thickness, levator ani thickness, urethral width, and intraprostatic urethral length. Our study is the first to establish that patients with complete ED exhibit a statistically significant increase in obturator internus thickness compared to those with preserved erectile function (*p* = 0.040; *p* < 0.05), as well as a significantly greater intraprostatic urethral length (*p* = 0.039; *p* < 0.05).

We created four nomograms with variable cut-off values based on the extent of nerve sparing and the surgical procedure used. These nomograms provide a tailored methodology for forecasting ED based on individualized patient treatment. In comparison to the initial nomogram established by Mulhall et al. [20], we noted that age, extent of nerve sparing, preoperative IIEF score, and comorbidity score are shared factors. Nonetheless, our analysis incorporates supplementary mpMRI parameters. Given its practical application, this model has the potential to aid clinicians in daily practice by facilitating individualized risk assessment of postoperative ED. This may thereafter empower both clinicians and patients to undertake suitable preventive or rehabilitative actions promptly.

## 5. Conclusions

Our study revealed that advanced age is a substantial risk factor for postoperative erectile dysfunction in patients receiving robot-assisted radical prostatectomy, and that bilateral nerve-sparing surgery is an advantageous method for preserving erectile function. The parameters we assessed, including surgical method, age, preoperative IIEF score, comorbidity index, obturator internus muscle thickness, and intraprostatic urethral breadth, demonstrated a statistically significant influence on erectile function. The nomogram we created offers individualized prognostic data to evaluate postoperative erectile function prior to surgery, based on these characteristics. Further large-scale, multi-center investigations are essential to enhance the accuracy and generalizability of these results and nomograms.

## Figures and Tables

**Table 1:** Demographic, preoperative and postoperative patient data.

	Complete Erectile Dysfunction (*n* = 73)	Preserved Erection (*n* = 38)	Total (*n* = 111)	*p*
Age (mean ± SD)	62.49 ± 6.42	58.66 ± 6.63	61.18 ± 6.72	^a^ 0.004
BMI (mean ± SD)	28 (20.1–39.0)	27.6 (20.8–32.4)	27.9 (20.1–39.0)	^b^ 0.914
Smoking	Non-smoker: 38 (52.1) Smoker: 15 (20.5) Ex-smoker: 20 (27.4)	Non-smoker: 17 Smoker: 11 Ex-smoker: 10	Non-smoker: 55 Smoker: 26 Ex-smoker: 30	^c^ 0.596
Charlson Comorbidity Index (mean ± SD)	4.15 ± 0.88	3.63 ± 0.85	3.97 ± 0.9	^a^ 0.004
Preoperative PSA (ng/dL) (mean ± SD)	7.1 ± 3.5	7.26 ± 3.65	7.16 ± 3.54	^a^ 0.808
Preoperative IIEF score (mean ± SD)	14.29 ± 5.34	20.16 ± 3.27	16.3 ± 5.49	^a^ 0.001
Biopsy pathology ISUP Grade 1 (%) ISUP Grade 2 (%) ISUP Grade 3 (%)	33 (45.2) 38 (52.1) 2 (2.7)	15 (39.5) 20 (52.6) 3 (7.9)	48 (43.2) 58 (52.3) 5 (4.5)	^c^ 0.455
Final pathology Gleason Grade Group 1 (%) Gleason Grade Group 2 (%) Gleason Grade Group 3 (%) Gleason Grade Group 4 (%) Gleason Grade Group 5 (%)	8 (11.0) 55 (75.3) 8 (11.0) 0 (0.0) 2 (2.7)	7 (18.4) 29 (76.3) 2 (5.3) 0 (0.0) 0 (0.0)	15 (13.5) 84 (75.7) 10 (9.0) 0 (0.0) 2 (1.8)	^c^ 0.430 ^c^ 0.430
Prostate volume (mL) (mean ± SD)	60.75 ± 32.34	54.59 ± 24.56	58.64 ± 29.93	^a^ 0.415

^a^ Student’s *t*-test; ^b^ Mann–Whitney U test; ^c^ Pearson Chi-Square test; BMI: body mass index; IIEF: International Index of Erectile Function; mL: milliliter; ng/mL: nanogram per milliliter; PSA: prostate-specific antigen; ISUP: International Society of Urological Pathology. Statistical test selection was based on normality testing (Shapiro–Wilk test) for continuous variables and expected cell frequencies for categorical variables.

**Table 2:** Multiparametric MRI measurements in different erectile function groups.

	Complete Erectile Dysfunction (*n* = 73)	Preserved Erection (*n* = 38)	Total (*n* = 111)	*p*
Membranous urethral length (mm)	15.6 (10.6–24.8)	14.2 (10.6–19)	15 (10.6–24.8)	^a^ 0.051
Membranous urethra–prostatic axis angle	56.6 (32.7–86.6)	55.6 (36.9–77.5)	56.3 (32.7–86.6)	^a^ 0.695
Obturator internus muscle thickness (mm)	20.3 (13–27.7)	19.8 (15.4–23.3)	20 (13–27.7)	^a^ 0.040
Levator ani muscle thickness (mm)	9.6 (6.3–14.9)	10.4 (6.8–12.7)	9,9 (6.3–14.9)	^a^ 0.305
Urethral width (mm)	10.5 (6.7–13.4)	10.4 (7.5–14.1)	10,4 (6.7–14.1)	^a^ 0.870
Intraprostatic urethral length (mm)	34.7 (19–57.6)	31.4 (21.8–51.5)	33,5 (19–57.6)	^b^ 0.039
MR AP pelvic diameter (mm)	124.9 (11.9–139.8)	124.2 (80.5–141.6)	124,9 (11.9–141.6)	^b^ 0.408
MR transverse pelvic diameter (mm)	116.5 (96.5–142.9)	115.6 (99–151.3)	116,3 (96.5–151.3)	^b^ 0.941

^a^ Mann–Whitney U test; ^b^ Student’s *t*-test; MR: magnetic resonance; AP: anterior–posterior. Statistical test selection was based on normality assessment using Shapiro-Wilk test for each variable. Variables with non-normal distribution were analyzed using the Mann–Whitney U test, while normally distributed variables were analyzed using Student’s *t*-test.

**Table 3:** Surgical techniques.

		Complete Erectile Dysfunction (*n* = 73)	Preserved Erection (*n* = 38)	Total (*n* = 111)	*p*
Surgical technique	Conventional	61 (83.6)	23 (60.5)	84 (75.7)	
	Modified	10 (13.7)	15 (39.5)	25 (22.5)	^a^ 0.005
	Retzius-sparing	2 (2.7)	0(0.0)	2 (1.8)	
Nerve-sparing technique	None Unilateral Bilateral	24 (32.9) 6 (8.2) 43 (58.9)	1 (2.6) 4 (10.5) 33 (86.8)	25 (22.5) 10 (9.0) 76 (68.5)	^b^ 0.001 ^b^ 0.001
Lymphadenectomy	None Yes	42 (57.5) 31 (42.5)	28 (73.7) 10 (26.3)	70 (63.1) 41 (36.9)	^b^ 0.094

^a^ Fisher–Freeman–Halton test; ^b^ Pearson Chi-Square test. The Fisher–Freeman–Halton test was used for surgical technique comparison due to cells with expected frequencies <5 in the contingency table. The Pearson Chi-Square test was used for other categorical variables where expected cell frequencies were adequate (≥5).

## Data Availability

All data analyzed in the present study are included in this article.

## Abbreviations

The following abbreviations are used in this manuscript:

AP	Anterior–Posterior
BMI	Body Mass Index
ED	Erectile Dysfunction
EF	Erectile Function
IIEF	International Index of Erectile Function
PDEI	Phosphodiesterase Inhibitor
PSA	Prostate-Specific Antigen
RARP	Robot-Assisted Radical Prostatectomy
RP	Radical Prostatectomy

## References

[1] Sung H., Ferlay J., Siegel R.L., Laversanne M., Soerjomataram I., Jemal A., Bray F. (2021). Global Cancer Statistics 2020: GLOBOCAN Estimates of Incidence and Mortality Worldwide for 36 Cancers in 185 Countries. CA Cancer J. Clin..

[2] Bergengren O., Pekala K.R., Matsoukas K., Fainberg J., Mungovan S.F., Bratt O., Bray F., Brawley O., Luckenbaugh A.N., Mucci L. (2023). 2022 Update on Prostate Cancer Epidemiology and Risk Factors—A Systematic Review. Eur. Urol..

[3] Stolzenburg J.U., Graefen M., Kriegel C., Michl U., Martin Morales A., Pommerville P.J., Manning M., Büttner H., Henneges C., Schostak M. (2015). Effect of surgical approach on erectile function recovery following bilateral nerve-sparing radical prostatectomy: An evaluation utilising data from a randomised, double-blind, double-dummy multicentre trial of tadalafil vs. placebo. BJU Int..

[4] Bratu O.G., Diaconu C.C., Mischianu D.L., Constantin T., Stanescu A.M., Bungau S.G., Ionita-Radu F., Marcu R.D. (2019). Therapeutic options in patients with biochemical recurrence after radical prostatectomy (Review). Exp. Ther. Med..

[5] Tal R., Alphs H.H., Krebs P., Nelson C.J., Mulhall J.P. (2009). Erectile function recovery rate after radical prostatectomy: A meta-analysis. J. Sex. Med..

[6] Mulhall J.P. (2009). Defining and reporting erectile function outcomes after radical prostatectomy: Challenges and misconceptions. J. Urol..

[7] Resnick M.J., Koyama T., Fan K.H., Albertsen P.C., Goodman M., Hamilton A.S., Hoffman R.M., Potosky A.L., Stanford J.L., Stroup A.M. (2013). Long-term functional outcomes after treatment for localized prostate cancer. N. Engl. J. Med..

[8] Johansson E., Steineck G., Holmberg L., Johansson J.E., Nyberg T., Ruutu M., Bill-Axelson A., SPCG-4 Investigators (2011). Long-term quality-of-life outcomes after radical prostatectomy or watchful waiting: The Scandinavian Prostate Cancer Group-4 randomised trial. Lancet Oncol..

[9] Kattan M.W., Vickers A.J., Yu C., Bianco F.J., Cronin A.M., Eastham J.A., Klein E.A., Reuther A.M., Edson Pontes J., Scardino P.T. (2009). Preoperative and post-operative nomograms incorporating surgeon experience for clinically localized prostate cancer. Cancer.

[10] Zelefsky M.J., Kattan M.W., Fearn P., Fearon B.L., Stasi J.P., Shippy A.M., Scardino P.T. (2007). Pretreatment nomogram predicting ten-year biochemical outcome of three-dimensional conformal radiotherapy and intensity-modulated radiotherapy for prostate cancer. Urology.

[11] Xu J., Dailey R.K., Eggly S., Neale A.V., Schwartz K.L. (2011). Men’s perspectives on selecting their prostate cancer treatment. J. Natl. Med. Assoc..

[12] Schroeck F.R., Krupski T.L., Sun L., Albala D.M., Price M.M., Polascik T.J., Robertson C.N., Tewari A.K., Moul J.W. (2008). Satisfaction and regret after open retropubic or robot-assisted laparoscopic radical prostatectomy. Eur. Urol..

[13] Kucuk E.V., Sobay R., Tahra A. (2023). Ultrapreservation in Robotic Assisted Radical Prostatectomy Provides Early Continence Recovery. JSLS.

[14] Brajtbord J.S., Punnen S., Cowan J.E., Welty C.J., Carroll P.R. (2014). Age and baseline quality of life at radical prostatectomy--who has the most to lose?. J. Urol..

[15] Alemozaffar M., Regan M.M., Cooperberg M.R., Wei J.T., Michalski J.M., Sandler H.M., Hembroff L., Sadetsky N., Saigal C.S., Litwin M.S. (2011). Prediction of erectile function following treatment for prostate cancer. JAMA.

[16] Salter C.A., Tin A.L., Bernie H.L., Nascimento B., Katz D.J., Benfante N.E., Carlsson S.V., Mulhall J.P. (2022). Predictors of Worsening Erectile Function in Men with Functional Erections Early After Radical Prostatectomy. J. Sex. Med..

[17] Seetharam Bhat K.R., Moschovas M.C., Sandri M., Reddy S., Onol F.F., Noel J., Rogers T., Schatloff O., Coelho R., Ko Y.H. (2021). Stratification of Potency Outcomes Following Robot-Assisted Laparoscopic Radical Prostatectomy Based on Age, Preoperative Potency, and Nerve Sparing. J. Endourol..

[18] Zhao Z., Zhu H., Yu H., Kong Q., Fan C., Meng L., Liu C., Ding X. (2017). Comparison of intrafascial and non-intrafascial radical prostatectomy for low risk localized prostate cancer. Sci. Rep..

[19] Pikramenos K., Zachou M., Papadopoulos D., Papatsoris A., Varkarakis I., Mitsogiannis I. (2023). Post Radical Prostatectomy Erectile Dysfunction. A Single Centre Experience. Cureus.

[20] Mulhall J.P., Kattan M.W., Bennett N.E., Stasi J., Nascimento B., Eastham J., Guillonneau B., Scardino P.T. (2019). Development of Nomograms to Predict the Recovery of Erectile Function Following Radical Prostatectomy. J. Sex. Med..

